# Anesthetic Considerations for Liver Transplantation in a Patient with Mitochondrial Neurogastrointestinal Encephalopathy Syndrome

**DOI:** 10.7759/cureus.5038

**Published:** 2019-06-29

**Authors:** Jai Madhok, Jason Leong, Jed Cohn

**Affiliations:** 1 Anesthesiology, Perioperative and Pain Medicine, Stanford University, Stanford, USA

**Keywords:** liver transplantation, mngie, anesthesia, perioperative management

## Abstract

Mitochondrial neurogastrointestinal encephalopathy (MNGIE) is a rare, complex mitochondrial disorder with variable phenotypes caused by a defect in the TYMP gene that codes for the thymidine phosphorylase enzyme. Orthotopic liver transplantation (OLT) has been proposed as a curative option for patients by using the liver as a source to restore thymidine phosphorylase levels in the body. Anesthetic considerations for this syndrome have not been clearly outlined in the past. We describe the clinical presentation of a young woman with MNGIE, her perioperative assessment, and intraoperative management during liver transplantation.

## Introduction

Mitochondrial neurogastrointestinal encephalopathy (MNGIE) is a rare, inherited disorder caused by defects in the TYMP gene that results in dysfunctional thymidine phosphorylase (TP). TP participates in nucleotide metabolism and dysfunction leads to an accumulation of its nucleoside substrates that disrupts mitochondrial DNA processing [1-2]. The estimated prevalence of MNGIE is approximately 1-9 per 1,000,000 and fewer than 200 cases have been described [3-4]. Diagnostic testing involves sequencing TYMP gene variations [5], measuring TP activity [2, 5], or measuring serum levels of thymidine [1-2, 5] and deoxyuridine [2, 4-5]. 

MNGIE is a progressive multisystem disorder that has a variable age of onset (first to fifth decade) and high mortality, commonly between 20 and 40 years of age [3]. Patients present with gastrointestinal (GI) manifestations secondary to widespread dysmotility [3] with symptoms including dysphagia, gastroesophageal reflux disease, gastroparesis, and intestinal pseudo-obstruction [3] all leading to severe cachexia. Sensorimotor neuropathy and myopathy (muscle weakness, ptosis, ophthalmoparesis) [3] may be present. Leukoencephalopathy, seen as diffuse loss of white matter on MRI, is frequently present [2-3, 5]. Due to mitochondrial DNA dysfunction, anaerobic pathways prevail, and patients exhibit chronic lactic acidosis that is exacerbated by glucose loads and other stressors [6]. Uncommonly, the patients may have cardiac symptoms such as prolonged QT interval, supraventricular tachycardia, and cardiac arrest [3].

Management of these patients involves supportive care (GI and neuropathic symptom control, nutritional optimization) and prevention of complications (aspiration, malnutrition, and deconditioning) [5]. Multiple strategies have been trialed to correct the underlying biochemical defect in MNGIE, but efficacy and outcomes are variable. Normalization of DNA substrates through the delivery of TP by platelet transfusions [7], erythrocyte-capsulated TP enzymes [8], or via direct removal of metabolites by dialysis [9] have not provided sustained clinical benefit. Allogeneic bone marrow transplantation (BMT) has been proposed as a treatment for MNGIE syndrome [7, 10] by effectively restoring endogenous TP enzyme transcription but it can be associated with high mortality [10]. An alternative approach to reinstituting endogenous TP supply using orthotopic liver transplantation (OLT) has been performed, based on high TP concentrations in hepatic tissues [11]. A 400-day follow-up of the index patient showed mild improvement in neurologic function [12]. 

To date, very few patients with MNGIE have undergone liver transplantation. We present the case of a 21-year-old woman with MNGIE undergoing OLT under general anesthesia. The patient has provided Health Insurance Portability and Accountability Act authorization to publish this case report. 

## Case presentation

A 21-year-old female (33.6 kg, 5 feet tall, BMI 14.4 kg/m2) with MNGIE presented for liver transplantation. Her calculated model for end-stage liver disease (MELD) score was 7, but she was listed at a MELD of 35 with exception points granted for her disorder.

Prior to diagnosis, she developed a constellation of symptoms including fatigue, nausea, vomiting, abdominal pain, anorexia, and paresthesias. Approximately one year prior to transplant, the diagnosis of MNGIE was made based on GI dysfunction, neurologic involvement (neuropathic pain, bilateral foot drop, leukoencephalopathy on MRI), and cachexia with severe muscle weakness. Genetic testing identified a novel TYMP mutation (homozygous for c.214+1G>T, intron 2, in canonical splice site). Thymidine levels in the plasma were markedly elevated at 3,095 nM (normal < 700 nM). The patient deteriorated despite maximal medical care and was largely wheelchair-dependent. Reflux and dysmotility were refractory necessitating total parenteral nutrition (TPN). Persistent lactic acidosis was present (baseline 3-4 mmol/L), and complications of chronic TPN included hyperglycemia with insulin resistance, cholestasis, and hepatosteatosis.

The preoperative evaluation was notable for hepatomegaly and severe steatosis on abdominal imaging. EKG was notable for sinus tachycardia. Echocardiography and cardiac MRI demonstrated left ventricular ejection fraction of 50% and mild myocardial fibrosis. Pulmonary function tests exhibited a restrictive defect consistent with neuromuscular weakness. Preoperative medications included acetaminophen, Vitamins B1, B12, and D, gabapentin, insulin, ondansetron, and simethicone. Given her underlying mitochondrial disease, chloramphenicol, nucleoside reverse transcriptase inhibitors, phenytoin, tetracyclines, and valproic acid were listed as allergies due to their inhibition of oxidative phosphorylation.

On arrival to the operating room, the patient’s TPN and insulin pumps were disconnected, and dextrose and insulin infusions were initiated. Her stomach was decompressed prior to induction using her in-situ gastrostomy tube. She received 2 mg of midazolam for anxiolysis and underwent rapid sequence induction with 50 mcg of fentanyl, 6 mg of etomidate, 20 mg of ketamine and 50 mg of rocuronium. Endotracheal intubation was uneventful. Vascular access was established according to our standard practice with a right femoral arterial line and two 9 Fr introducer sheaths in the left internal jugular vein (for fluid administration and pulmonary artery catheter placement). An orogastric tube was placed for gastric decompression. Transesophageal echocardiography was utilized for hemodynamic assessment. A maintenance anesthetic of O2-air-isoflurane (0.5-1%) mixture was titrated to a patient state index < 50 using processed EEG with SEDLine^TM ^monitoring (Masimo, Irvine, CA). Octreotide infusion was started prior to incision at 2 mcg/kg^-1^/hr^-1^(67.6 mcg/hr) for splanchnic vasoconstriction to reduce portal venous blood flow and minimize blood loss during transplantation. Glucose and potassium management (goal pre-reperfusion serum potassium < 4 mEq/L) was done with simultaneous infusions of dextrose 20% (10-50 ml/hr) and insulin (3-12 units/hr). Electrolytes and acid-base parameters were managed with boluses of sodium bicarbonate, calcium chloride, and insulin/dextrose. The patient responded appropriately to these interventions and no exaggerated or refractory metabolic derangements were observed.

OLT was conducted using venovenous bypass (right internal jugular and left femoral venous cannulation) as is standard for our institution. Methylprednisolone (175 mg) was given during the anhepatic phase for immunosuppression. The patient tolerated reperfusion well without significant hemodynamic distress. She received a total of 50 mg (1.5 mg/kg) of ketamine and 350 mcg (10 mcg/kg) of fentanyl during the seven-hour case. The fluid balance included input of 1.5 L Normosol-R, 1 L of 5% albumin, with 700 cc of urine output and an estimated blood loss of 1 L. No blood products were transfused. The patient remained electively intubated and was transferred to the ICU on infusions of dexmedetomidine (0.6 mcg/kg^-1^/hr^-1^) for sedation and phenylephrine (0.5 mcg/kg^-1/^min^-1^) for post-reperfusion vasoplegia (cardiac output 9.5 L/min, cardiac index 7.8 L/min^-1/^m^2^, SVR 589 dynes-sec/cm^5^). The patient was extubated on postoperative day (POD#) 0, and anti-thymocyte globulin (ATG) and tacrolimus were initiated for immunosuppression. Mycophenolate mofetil was added on POD #2 and she was transferred out of the ICU on POD #3. Her hospital course was complicated by mild T-cell mediated rejection necessitating a steroid taper and additional doses of ATG; MSSA bacteremia; and low-grade EBV and CMV viremia. The patient’s G-tube was converted to a gastrostomy-jejunostomy (G-J) tube. She was discharged on POD #39 able to tolerate partial enteral feeds but continued to require TPN supplementation. Surveillance labs demonstrated normalization of thymidine levels within 30 days post-OLT. 

The patient, over one-year post-transplantation, is now able to walk better and has improved motor strength. She is also able to eat one meal per day, though still continues on cyclical TPN for nutritional support. There is no evidence of rejection on most recent liver biopsy.

## Discussion

MNGIE is a rare disease with high mortality and limited treatment options. Liver transplantation has been proposed as a potentially curative option [11], and there are reports of its efficacy [12-13]. For the anesthesiologist, MNGIE creates clinical and biochemical challenges that necessitate careful planning. To our knowledge this is the first report detailing the intraoperative anesthetic management of a patient with MNGIE undergoing liver transplantation.

In addition to comprehensive preoperative cardiopulmonary evaluation, the metabolic profile of these patients should be studied paying special attention to lactate and glucose levels. Lactic acidosis and unpredictable glucose sensitivity warrants frequent glucose monitoring, and preoperative fasting should be minimized or replaced by intravenous dextrose. Many physiologic and metabolic stressors (hypo/hyperthermia, hypoxia, hypotension) can worsen lactic acidosis and should be managed diligently [14]. 

Exposure to inhibitors of oxidative phosphorylation should be minimized as in other mitochondrial disorders. While all volatile and intravenous anesthetic agents are shown to inhibit the electron transport chain (ETC), short-term exposure is tolerated well in most patients [14-15]. Extended infusions of propofol, given its multiple sites of ETC inhibition, should be avoided [14]. There is no documented risk of malignant hyperthermia with MNGIE. Local anesthetics such as bupivacaine and ropivacaine can impair ETC function [14] and should be used with caution, particularly in the presence of cardiomyopathy. We showed that under close monitoring use of volatile anesthetics, non-depolarizing neuromuscular blocking agents, and corticosteroids for immunosuppression was safe in MNGIE.

Due to increased anesthetic sensitivity, hypnotic depth monitoring may minimize the effects of anesthetic overdose on neurocognitive and hemodynamic status. Pharmacokinetics and pharmacodynamics of drugs may be altered due to extremely low body weight and lean body mass. Strict aspiration precautions should be employed given severe reflux and dysmotility. Respiratory insufficiency secondary to muscle weakness may increase the risk of postoperative pulmonary complications. Neuromuscular blockade should be minimized and fully reversed prior to extubation, with avoidance of depolarizing agents to avoid an exaggerated hyperkalemic response. Lastly, given severe cachexia, invasive devices may need to be downsized, and bedside ultrasonography can aid in selecting optimal size and location for invasive access.

This case is noteworthy as it presents a unique indication for liver transplantation in the absence of end-stage liver failure. Further, it is one of the few instances where the complexities of mitochondrial dysfunction are subjected to the magnitude of physiologic stressors of liver transplantation. With careful preoperative assessment and comprehensive intraoperative management, the patient had a successful perioperative outcome.

## Conclusions

MNGIE is a rare disorder of TP dysfunction that results in the accumulation of unmetabolized thymidine leading to mitochondrial dysfunction. The disorder is characterized by profound gastrointestinal dysfunction, neurologic impairment, and metabolic derangements. We demonstrate that anesthesia for a major procedure can be safely conducted in the MNGIE patient by adhering to mitochondrial precautions for anesthesia coupled with diligent management of the multisystem manifestations of the disease. 

## References

[1] Spinazzola A, Marti R, Nishino I (2002). Altered thymidine metabolism due to defects of thymidine phosphorylase. J Biol Chem.

[2] Hirano M, Nishigaki Y, Marti R (2004). Mitochondrial neurogastrointestinal encephalomyopathy (MNGIE): a disease of two genomes. Neurologist.

[3] Garone C, Tadesse S, Hirano M (2011). Clinical and genetic spectrum of mitochondrial neurogastrointestinal encephalomyopathy. Brain.

[4] Yadak R, Sillevis Smitt P, van Gisbergen MW, van Til NP, de Coo IFM (2019). Mitochondrial neurogastrointestinal encephalomyopathy caused by thymidine phosphorylase enzyme deficiency: from pathogenesis to emerging therapeutic options. Front Cell Neurosci.

[5] Hirano M (2016). Mitochondrial Neurogastrointestinal Encephalopathy Disease.

[6] Bardosi A, Creutzfeldt W, DiMauro S (1987). Myo-, neuro-, gastrointestinal encephalopathy (MNGIE syndrome) due to partial deficiency of cytochrome-c-oxidase. A new mitochondrial multisystem disorder. Acta Neuropathol.

[7] Lara MC, Weiss B, Illa I (2006). Infusion of platelets transiently reduces nucleoside overload in MNGIE. Neurology.

[8] Moran NF, Bain MD, Muqit MM, Bax BE (2008). Carrier erythrocyte entrapped thymidine phosphorylase therapy for MNGIE. Neurology.

[9] Yavuz H, Ozel A, Christensen M (2007). Treatment of mitochondrial neurogastrointestinal encephalomyopathy with dialysis. Arch Neurol.

[10] Halter JP, Michael W, Schupbach M (2015). Allogeneic haematopoietic stem cell transplantation for mitochondrial neurogastrointestinal encephalomyopathy. Brain.

[11] Boschetti E, D’Alessandro R, Bianco F (2019). Liver as a source for thymidine phosphorylase replacement in mitochondrial neurogastrointestinal encephalomyopathy. PLoS One.

[12] De Giorgio R, Pironi L, Rinaldi R (2016). Liver transplantation for mitochondrial neurogastrointestinal encephalomyopathy. Ann Neurol.

[13] D'Angelo R, Rinaldi R, Pironi L (2017). Liver transplant reverses biochemical imbalance in mitochondrial neurogastrointestinal encephalomyopathy. Mitochondrion.

[14] Niezgoda J, Morgan PG (2013). Anesthetic considerations in patients with mitochondrial defects. Paediatr Anaesth.

[15] Wallace JJ, Perndt H, Skinner M (1998). Anaesthesia and mitochondrial disease. Paediatr Anaesth.

